# Drought stress and changes in the lignin content and composition in *Eucalyptus*

**DOI:** 10.1186/1753-6561-5-S7-P103

**Published:** 2011-09-13

**Authors:** Jullyana Moura-Sobczak, Uiara Souza, Paulo Mazzafera

**Affiliations:** 1 1. Departamento de Fisiologia Vegetal. Instituto de Biologia - Unicamp. CEP: 13083-970 Campinas, SP, Brazil

## 

It is known that many abiotic stresses, such as mineral deficiency, drought, UV-B radiation, wind and low temperatures, alter the quantity and composition of lignin in several species [1]. The aim of this work was to verify if the drought stress may cause changes in the quantity and composition of lignin in *Eucalyptus globulus* Labill and in the hybrids *E. urograndis* (*E. urophylla* x *E. grandis*) and *E. uroglobulus* (*E. globulus x E. urograndis*). In the experiments the plants were divided in three groups (control, drought and drought recovered.) The control plants were irrigated daily. The plants from the group “drought” were not irrigated and were collected when wilt symptom was observed. The plants from the group “drought recovered” were irrigated when wilt was observed and were collected after recovery.

Samples of basal and apical regions of the stem were collected and analyzed for total lignin with thioglycolic acid [2] and analyzed by GC-MS to determine lignin monomeric composition [3].

*E. urograndis* subjected to drought decreased the amount of lignin in the stem apical regions and increased lignin in the basal region. *E. globulus* showed opposite behavior in apical regions and showed no significant changes in the basal regions. *E. uroglobulus* showed a pattern similar to *E. urograndis* in apical regions and similar to E*. globulus* in basal regions.

Although *E. urograndis* and *E. uroglobulus* reduced lignin and *E. globulus* increased in the apical part of the stem, it was observed that these different adjustments of lignin deposition eventually result in an increased proportion of S/G in both species. Moreover, when the amount of lignin is increased in the basal regions of *E. urograndis* there is a decrease in the proportion S/G.

Increasing the proportion S/G, either by increasing the amount of lignin-rich syringyl units or reduction of coniferyl units can be an important aspect in the adaptation of both species to drought stress.
